# Vaccine Incentives Harm Intrinsic Motivation: Evidence From a Priming Experiment

**DOI:** 10.1002/hec.70061

**Published:** 2025-11-11

**Authors:** Johnny Huynh, Corey Jacinto, James Huynh

**Affiliations:** ^1^ Dartmouth College Hanover New Hampshire USA; ^2^ University of California Los Angeles Los Angeles California USA; ^3^ University of Michigan Ann Arbor Michigan USA

## Abstract

Monetary incentives for vaccination may undermine intrinsic motivation, but evidence on this effect remains scarce. We conducted an experiment among 513 vaccine‐hesitant adults to test whether priming individuals with a monetary incentive reduces their willingness to vaccinate against COVID‐19. Our findings show that one in seven were willing to vaccinate without an incentive but declined the vaccine when asked to consider a payment. Additionally, priming participants lowered their perceptions of vaccine safety by 9 pp and prosocial attitudes toward vaccination by 10 pp. These negative effects were concentrated among men, racial and ethnic minorities, and participants with lower preexisting trust in the vaccine. Our results highlight an unintended consequence of vaccine incentives.

## 1 Introduction

Dating back to Pigou's (1920) seminal work on externalities, economists have viewed monetary incentives as a tool for promoting behaviors that generate positive spillover effects. For instance, governments worldwide have used conditional cash transfers to encourage investments in health and human capital.^1^ More recently, during the early years of the COVID‐19 pandemic, when vaccine uptake in the U.S. fell short of expectations, policymakers proposed paying vaccine‐hesitant adults to get vaccinated, arguing that the public health benefits outweighed the costs. Notably, President Biden urged state and local governments to “provide $100 payments for every newly vaccinated American, as an extra incentive to boost vaccination rates, protect communities, and save lives” (Reuters 2021). Mankiw (2020) also described vaccine incentives as “textbook economics.”^2^ However, critics warned that monetary incentives could backfire by crowding out intrinsic motivation, as observed in other contexts where payments displaced prosocial behavior, such as blood donation.^3^ Are vaccine incentives effective at encouraging the vaccine‐hesitant to get vaccinated?

The central challenge in addressing this question is that vaccine incentives induce competing effects. The price effect makes vaccination more attractive by effectively lowering its marginal cost. In contrast, the psychological effect, in which a monetary incentive crowds out intrinsic motivation through various behavioral channels, can deter individuals from getting vaccinated (Ariely et al. 2009; Bowles and Polania‐Reyes 2012). While economic theory clearly outlines these opposing forces, few studies have quantified the magnitude of the psychological effect among vaccine‐hesitant adults. Nor are the mechanisms behind this effect well understood. To address this gap, we conducted a survey experiment designed to disentangle the price effect and psychological effect, during a period of declining demand for COVID‐19 vaccines. Our experiment employed a behavioral priming technique to isolate the psychological impact of introducing a monetary incentive. Additionally, we developed an econometric model of treatment take‐up, inspired by Heckman and Pinto (2021), to characterize the subgroup of individuals for whom the idea of an incentive discourages vaccination.

In our experiment, 513 vaccine‐hesitant adults were randomly assigned to one of two experimental arms. Participants in the control arm were asked to consider getting vaccinated without any mention of incentives, while those in the incentive arm were asked to consider vaccination at varying payment levels, including zero dollars. Our primary outcome is participants' stated willingness to get vaccinated against COVID‐19, while our secondary outcomes are their perceptions of vaccine safety and whether they view vaccination as a social responsibility. The logic of our experiment is straightforward: if monetary incentives operate solely by reducing the marginal cost of vaccination, then offering zero dollars should yield the same willingness to vaccinate as the control arm. Any deviation from this parity would indicate the presence of a psychological effect.

Our findings are both novel and striking. We estimate that monetary incentives deterred 14% of participants from vaccination; these individuals would have considered getting vaccinated when no payment was mentioned (control arm) but opted out when a reward was introduced (incentive arm). This subgroup was more likely to consist of men from racial and ethnic minority backgrounds, a demographic that has exhibited higher levels of medical mistrust in prior research on vaccine uptake in the U.S. (Thompson et al. 2021). Additionally, deterred individuals expressed, at baseline, greater skepticism about the vaccine's safety, indicating that incentives interact with preexisting levels of trust in an intervention's safety rather than uniformly increasing or decreasing uptake.

Through what channels do monetary incentives discourage vaccination? One explanation is that an incentive signals to participants that the vaccine has undesirable traits. For instance, when financial compensation is offered, individuals may interpret it as a signal that the vaccine is unsafe. Another explanation is that monetary incentives undermine prosocial motives for vaccination by shifting the perceived locus of control from internal to external (Frey and Oberholzer‐Gee 1997). We find evidence for both channels. Participants in the incentive arm were 9% points more likely to perceive vaccines as unsafe and 10% points more likely to doubt the social responsibility of vaccination, compared to the control arm. We then conducted a mediation analysis, estimating that one‐quarter of the psychological effect induced by an incentive can be attributed to negative shifts in individuals' perceptions of the vaccine.

Finally, after establishing that monetary incentives deterred 14% of participants from vaccination, we recruited a subset of 27 participants for qualitative interviews. We designed a semi‐structured protocol to elicit beliefs about participants' decisions on whether to get vaccinated, their risk perceptions, and their responses to real and hypothetical government policies. The qualitative interviews corroborate that monetary incentives heightened suspicion regarding vaccine effectiveness and safety among vaccine‐hesitant adults. These rich qualitative data not only provide context for our causal findings but also allow us to systematically identify themes driving vaccine hesitancy within our sample.

Our paper makes two key contributions. First, these findings advance our understanding of the determinants of vaccine demand and the impact of monetary incentives on this demand. This topic is crucial for policymakers aiming to reduce morbidity from COVID‐19 and other infectious diseases, especially given the recent decline in measles, mumps, and rubella vaccine coverage in the U.S. (Seither et al. 2024). Many other studies have evaluated the effectiveness of vaccine incentives, but this literature has produced conflicting findings, with estimates ranging from null (Kreps et al. 2021; Lang et al. 2023; Law et al. 2022; Thirumurthy et al. 2022), to positive (Barber and West 2022; Klüver et al. 2021; Serra‐Garcia and Szech 2023), and even negative (Zhang and Lane 2023).^4^ For example, two “gold standard” randomized controlled trials testing similarly‐sized COVID‐19 vaccine incentives reached very different conclusions about their efficacy (Campos‐Mercade et al. 2021; Chang et al. 2021). When do incentives succeed, and when do they fail? Our paper helps reconcile the conflicting findings in this literature by demonstrating that the effect of a vaccine incentive on uptake is heterogeneous. Incentives are less effective in populations with a higher share of deterred individuals. We developed a procedure to characterize deterred individuals using data from our experiment, providing researchers and policymakers a tool to identify subpopulations that may be more resistant to monetary incentives.

Our study also adds to the growing body of mega‐study evidence on interventions to increase vaccine uptake, including large‐scale field trials that tested text‐message reminders and monetary incentives (Milkman et al. 2021, 2022, 2024). While those studies demonstrated the potential of nudges to increase vaccination rates, subsequent work has highlighted their limitations, including modest effect sizes and heterogeneity across populations (DellaVigna and Linos 2022; Szaszi et al. 2018). Our findings highlight a less‐explored source of heterogeneity: incentives can crowd out intrinsic motivation, with one in seven vaccine‐hesitant adults in our study deterred by the idea of payment. We build on this literature by clarifying why incentive‐based interventions may succeed in some contexts but backfire in others.

Finally, our paper makes a methodological contribution by integrating models of intrinsic motivation (Bénabou and Tirole 2003, 2006; Titmuss 1970; Laffont and Martimort 2001; Gneezy et al. 2011; Bowles and Polania‐Reyes 2012) with econometric tools for analyzing treatment‐response types (Heckman and Pinto 2021; Pinto 2021; Mogstad et al. 2018). Monetary incentives induce two competing effects: while the price effect makes the incentivized action more attractive, the psychological effect works in the opposite direction. In the context of our experiment, we formalized a monotonicity condition to point identify the share of individuals for whom the psychological effect is nonzero. We also show that, due to these opposing forces, the never‐taker share is only partially identified. We estimated sharp bounds for this share, drawing on insights from Navjeevan et al. (2023). Finally, we estimated a mediation model demonstrating that vaccine incentives weaken perceptions of vaccine safety and prosocial attitudes toward vaccination, providing empirical evidence that these perceptions mediate incentives' negative psychological effects.

The remainder of the paper is organized as follows. Section 2 provides background information on vaccine hesitancy, including a theoretical framework. Section 3 describes our survey experiment, while Section 4 develops the econometric model to analyze the experimental data. Section 5 presents our main results, and Section 6 examines potential mediators for our results. Section 7 evaluates our qualitative evidence. Section 8 concludes.

## 2 Background and Theory

### 2.1 The COVID‐19 Pandemic

By the end of 2021, the COVID‐19 pandemic had claimed 462,000 lives and caused $4.8 trillion in economic losses in the U.S. (Ahmad et al. 2022; Walmsley et al. 2021). The pandemic's toll spurred an unprecedented effort to accelerate vaccine development through public‐private partnerships. Operation Warp Speed, for example, united federal agencies with pharmaceutical companies to expedite vaccine research, development, and distribution.^5^ In December 2020, the U.S. Food and Drug Administration granted emergency‐use authorization for the Pfizer‐BioNTech and Moderna mRNA vaccines, followed by the Johnson & Johnson vaccine in February 2021. These emergency‐use authorizations were issued based on less evidence than is typically required under standard approval processes. This raised concerns among skeptics regarding long‐term safety and efficacy. Following emergency‐use authorization, vaccine doses were allocated to states on a per capita basis, leaving each state responsible for its own rollout. Although public health guidelines recommended prioritizing health care personnel and long‐term care facility residents for the initial doses, states varied in their prioritization strategies.

By July 2021, 67% of U.S. adults had received at least one dose of the COVID‐19 vaccine. Widespread vaccination was associated with declining infection rates nationwide, but regions with below‐average uptake experienced surges in cases due to the highly transmissible Delta variant. This resurgence prompted some employers and government agencies to enact vaccine mandates to increase coverage. Economic models underscored the importance of widespread vaccination in reducing uncertainty, lowering morbidity, and accelerating economic recovery (Eichenbaum et al. 2021). However, vaccine hesitancy emerged as a barrier to achieving herd immunity. Early surveys identified several key drivers of hesitancy, including misinformation, political polarization, and mistrust in the medical system, particularly among marginalized groups (Loomba et al. 2021; Sallam 2021).

**FIGURE A1:**
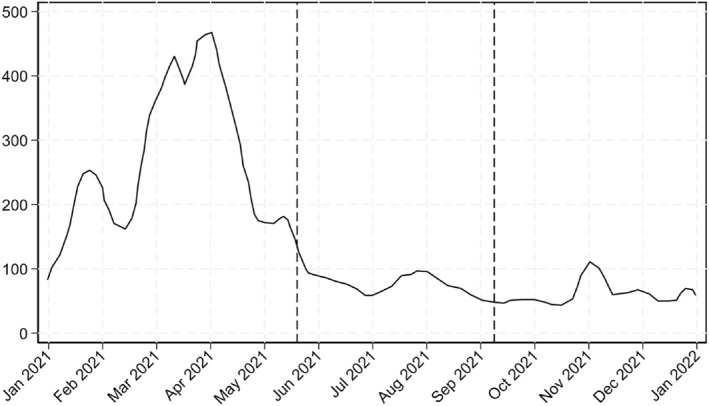
Weekly COVID‐19 vaccinations for age 5+ in L.A. County (in 1,000s). This figure presents weekly vaccination counts for Los Angeles County residents (ages 5+) who received at least one dose of a COVID‐19 vaccine. Data are sourced from the California Immunization Registry and include only individuals with valid residential addresses in Los Angeles County. Population estimates used to calculate vaccination rates are derived from the County of Los Angeles, Internal Services Department, Information Technology Service, Urban Research‐GIS Section. The x‐axis represents time from January 2021 to January 2022, while the y‐axis represents the number of weekly vaccinations administered. Dashed vertical lines indicate the time period in which we conducted our survey experiment (May 18, 2021 to September 8, 2021).

Los Angeles County, the setting of our experiment, faced many challenges in its COVID‐19 vaccination efforts, mirroring national trends in early to mid‐2021. Initially, vaccine distribution prioritized health care workers and residents of long‐term care facilities. By April 2021, all individuals aged 16 and older became eligible. Despite an aggressive county‐wide vaccination campaign, only 60% of eligible individuals had received at least one dose by July 2021, partly due to lower uptake among Black and Hispanic communities in the region (Saluja et al. 2021). Figure A1 plots weekly vaccinations in Los Angeles County throughout 2021 and highlights a sharp decline in demand beginning in April 2021. During this period, the county reported over 1000 new COVID‐19 cases per day. In response, Los Angeles County implemented a range of interventions to address disparities and increase uptake, including mobile vaccination clinics, community outreach programs, and multilingual public information campaigns. However, these efforts had only modest success, as vaccine demand remained low.

### 2.2 Theoretical Framework

We outline a framework to examine how a monetary incentive influences an individual's willingness to get vaccinated. In the absence of any incentive, an individual i’s baseline utility from vaccination is given by:

(1) $$U_{i}\left(0\right)=v_{i}-C_{i}$$

where $v_{i}$ represents i’s private valuation of vaccination and $C_{i}$ denotes the cost. Now, consider an incentive D>0, which modifies utility through two channels. The first is the price effect, represented by α>0, which captures the responsiveness of utility to the incentive by effectively lowering its marginal cost. The second is the psychological effect, which reduces intrinsic motivation through two behavioral mechanisms: risk signaling and undermining prosocial motives.

The risk‐signaling mechanism draws from signaling theory (Spence 1978), in which individuals interpret a monetary incentive as compensation for hidden risks, leading to a disutility term, $\rho R_{i}\left(D\right)$. The undermining of prosocial motives is rooted in self‐determination theory (Deci et al. 1999; Frey and Oberholzer‐Gee 1997), in which a monetary incentive shifts the perceived locus of control from internal to external, represented by $\delta M_{i}\left(D\right)$. Both $R_{i}\left(\cdot \right)$ and $M_{i}\left(\cdot \right)$ are expressed in utility terms. The resulting net utility from vaccination is given by:

(2) $$U_{i}\left(D\right)=v_{i}+\alpha D-\left[\rho R_{i}\left(D\right)+\delta M_{i}\left(D\right)\right]-C_{i}$$

where vaccination occurs if $U_{i}\left(D\right)\geq 0$. The minimum incentive required for individual i to vaccinate, denoted by $D_{i}^{*}$, satisfies:

(3) $$\alpha D_{i}^{*}-\left[\rho R_{i}\left(D_{i}^{*}\right)+\delta M_{i}\left(D_{i}^{*}\right)\right]=C_{i}-v_{i}$$

In the absence of any psychological effect, this threshold simplifies to:

(4) $$D_{i}^{*}=\frac{C_{i}-v_{i}}{\alpha }$$

However, when adverse psychological effects are present, the net impact of an incentive becomes ambiguous, as it may simultaneously encourage and discourage vaccination depending on the relative magnitudes of α, ρ, and δ. In an extreme case where $R_{i}\left(\cdot \right)$ or $M_{i}\left(\cdot \right)$ are steep, $D_{i}^{*}$ can approach infinity.

Each parameter in this theoretical framework has an empirical analog in our experiment. The valuation of vaccination $\left(v_{i}\right)$ reflects perceived private benefits of immunization, as proxied by respondents' beliefs about vaccine efficacy. The risk‐signaling parameter (ρ) reflects concerns that incentives imply hidden risks, which we capture through measures of safety perceptions. Finally, the prosocial‐motivation parameter (δ) represents whether respondents described vaccination as a social responsibility, a channel we measure explicitly for our mediation analysis. Thus, while stylized, the model's parameters map onto the psychological constructs that we directly measure and test for using experimental data.

## 3 Experimental Design and Data

### 3.1 Priming Experiment

**FIGURE A2:**
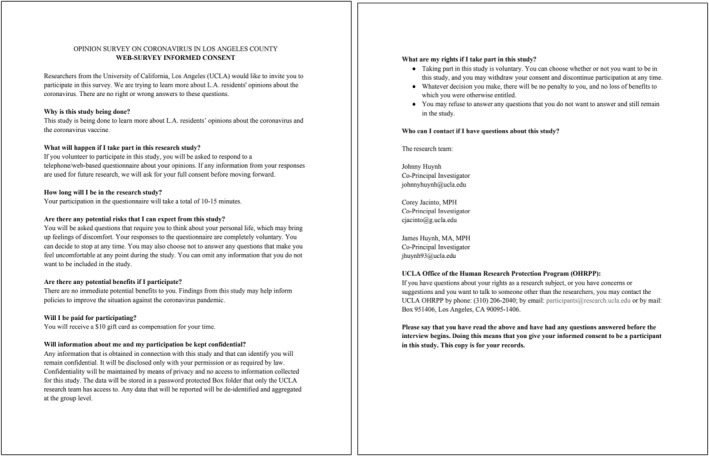
Informed consent documents.

**FIGURE A3:**
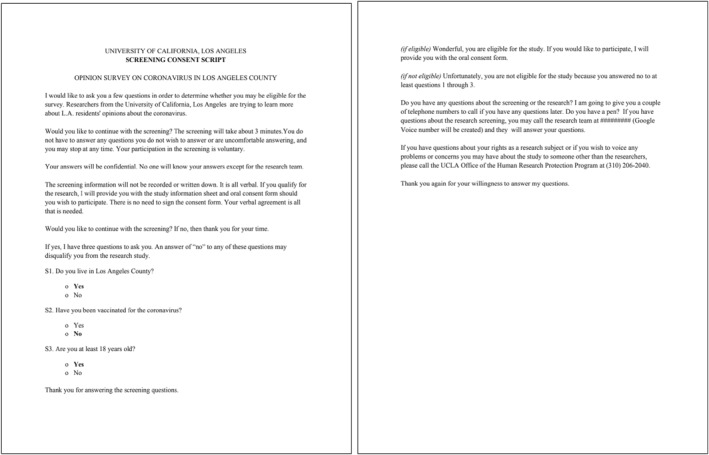
Screening consent documents.

We conducted a survey experiment to examine the decision to get vaccinated against COVID‐19 during a period of declining vaccine demand. To do this, we recruited adult participants through Los Angeles County community‐based organizations and Qualtrics between May 18 and September 8, 2021.^6^ We aimed to achieve a sample composition similar to Los Angeles County's racial and ethnic distribution while also oversampling non‐White racial groups by 10% points to ensure sufficient power for subgroup analyses. After obtaining informed consent, respondents completed a series of eligibility screening questions based on residence (Los Angeles County), age (18 or older), and vaccination status (not yet vaccinated). See Figures A2 and A3. Those who had received one dose of a two‐dose vaccine series were considered vaccinated. The baseline survey collected demographic information (age, gender, and race/ethnicity), socioeconomic information (household size, educational attainment, and income), self‐assessed health, attitudes toward health care professionals, and intention to receive the COVID‐19 vaccine. The survey was made available in English, Spanish, Korean, Tagalog, and Vietnamese.

Participants who responded “no” or “not sure” regarding their intention to receive the COVID‐19 vaccine were directed to the experimental module. We applied 1:1 simple randomization to assign participants to one of two treatment arms.^7^ Those assigned to the control arm received the following prompt: “Some officials have proposed letting any person get vaccinated for the coronavirus at no cost. In this scenario, would you consider getting vaccinated this week at no cost? [yes/no]”^8^ Although vaccines were already freely available, framing the control question this way helped preempt misconceptions about cost, an issue raised by one‐quarter of respondents in the baseline survey.

Participants assigned to the incentive arm were sequentially asked four questions about their willingness to get vaccinated under different monetary incentives:

1. Some officials have proposed paying people to get vaccinated for the coronavirus. In this scenario, would you consider getting vaccinated this week if you were offered $500? [yes/no]”
2. Would you consider getting vaccinated this week if you were offered $100? [yes/no]”
3. Would you consider getting vaccinated this week if you were offered $50? [yes/no]”
4. Would you consider getting vaccinated this week if you were not offered any payment? [yes/no]”

These hypothetical values were selected based on real‐world proposals from U.S. policymakers and employers (Litan 2020; U.S. Chamber of Commerce 2021). The questions' phrasing draws on priming methodologies commonly used in behavioral psychology research (Vohs et al. 2006). The final question in the incentive arm was designed to prime participants to think about monetary incentives (after internalizing the previous three questions) while simultaneously eliciting their willingness to vaccinate in the absence of any payment. Our primary outcome is an individual's stated willingness to consider getting vaccinated.

Note that priming individuals with any monetary incentive naturally introduces an anchoring bias, where valuations are strongly influenced by the initial payment (Lieder et al. 2018). Consequently, estimates from our experiment ought to be interpreted with $500 as the anchoring value.^9^ We deliberately did not vary this anchoring value because our sample size was not sufficiently powered to jointly test for both the priming and anchoring effects. We discuss the potential impact of anchoring bias in Section 5.3.

The survey design was intended to generate empirical analogs to the parameters from our theoretical framework. In particular, the framework suggests that the adverse psychological effect of an incentive may operate by shaping beliefs about vaccine safety (ρ) and prosocial responsibility (δ). To test these channels empirically, we embedded measures of perceived safety and prosocial attitudes within the survey experiment.

After being exposed to either the control or incentive module, we measured participants' perceptions of vaccine safety (“Do you believe the coronavirus vaccine is safe? [yes/no/not sure]”) and their prosocial attitudes toward vaccination (“Do you believe people have a responsibility to get vaccinated for the coronavirus? [yes/no/not sure]”).^10^ Because these questions assess beliefs rather than intentions, respondents were given the option to select “not sure.” The responses to these questions were used to evaluate the behavioral mechanisms underlying intrinsic motivation to get vaccinated and explore their interaction with monetary incentives. Thus, our secondary outcomes are individuals' perceptions of vaccine safety and their prosocial attitudes toward vaccination.

Nearly all respondents (98%) who reached the experimental module completed the survey. The survey instruments are provided in the Online Appendix. Respondents received a $10 Target gift card as compensation, delivered by mail or email according to their preference, and were invited to participate in a follow‐up qualitative interview for an additional $20 gift card. These qualitative interviews, conducted by video call or telephone within 3 months of survey completion, were recorded, transcribed into English, and analyzed by one of five trained analysts. Details of the qualitative data collection and analysis are presented in Section 7. The study was approved under UCLA IRB protocol #21‐000219.

### 3.2 Summary Statistics and Balance

**FIGURE A4:**
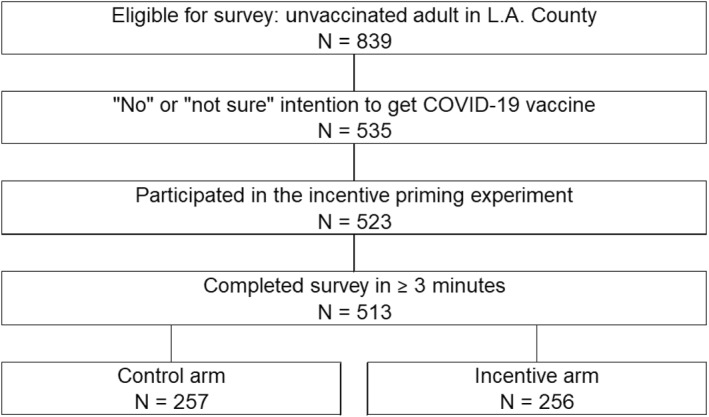
Participant flow diagram for the COVID‐19 vaccine incentive experiment. The flowchart illustrates the recruitment and randomization process for the survey experiment. Out of 839 unvaccinated adults in Los Angeles County who were eligible for the survey, 535 reported being either unwilling or uncertain about receiving the COVID‐19 vaccine and proceeded to the experimental module. 523 had completed the experimental module. Ten respondents who completed the survey in under 3 min were excluded. Participants were then randomly assigned in a 1:1 ratio to either the control group (*N* = 257) or the incentive group (*N* = 256).

A total of 513 individuals participated in the experiment.^11^ Figure A4 illustrates our sample selection process. Table 1 presents summary statistics. On average, participants were 34 years old; 38% identified as Latino, 35% as Black, 34% as White, and 9% as Asian. Half of the sample reported an annual household income below $40,000. One in eight participants had previously tested positive for COVID‐19, and 15% anticipated contracting either a mild or severe case within the next 3 months.

**TABLE 1:** Baseline statistics for participant characteristics by treatment arm.

	(1)	(2)	(3)	(4)
	Full sample	Control arm	Incentive arm	p‐value
Household size	3.44	3.57	3.32	0.13
Age	33.9	33.6	34.2	0.61
White male	0.13	0.14	0.12	0.52
White female	0.21	0.20	0.23	0.51
Non‐white male	0.21	0.20	0.23	0.34
Non‐white female	0.43	0.44	0.42	0.68
College graduate	0.32	0.30	0.33	0.55
Household income				
Less than $20,000	0.26	0.22	0.30	0.04
$20,000 to $39,999	0.23	0.26	0.21	0.15
$40,000 to $59,999	0.17	0.20	0.15	0.21
$60,000 to $79,999	0.11	0.11	0.10	0.58
$80,000 to $99,999	0.09	0.09	0.09	0.89
Greater than $100,000	0.14	0.12	0.16	0.19
Ever tested positive for COVID	0.12	0.13	0.11	0.50
Best guess for COVID risk				
None	0.85	0.83	0.86	0.28
Mild case	0.12	0.14	0.09	0.10
Serious case	0.04	0.03	0.04	0.48
Vaccine concerns (1–5 scale)				
Side effects	4.14	4.12	4.16	0.70
Not effective	3.71	3.76	3.65	0.24
Causes infection	3.40	3.46	3.34	0.32
Affordability	2.58	2.65	2.50	0.22
Accessibility	2.74	2.8	2.7	0.33
Compulsion by law	3.80	3.79	3.81	0.86
Compulsion by work/school	3.74	3.81	3.67	0.20
Observations	513	257	256	

*Note:* This table presents mean values for baseline variables, which were measured prior to the experimental module in order to assess balance across the control arm and incentive arm. Variables measure participants' demographics and pre‐treatment concerns about the COVID‐19 vaccine, with descriptions available in the survey instrument in the Online Appendix. Vaccine concern variables were measured on a 1‐5 point Likert scale, with higher values indicating greater concern regarding vaccine safety, efficacy, or compulsion. Column (1) reports mean values for the full sample, while columns (2) and (3) present mean values for participants assigned to the control arm and incentive arm, respectively. Column (4) reports two‐sided, single‐hypothesis p‐values from tests of mean equality between the control and incentive arms.

We also document substantial misinformation about vaccine eligibility among individuals in our sample, a pattern consistent with prior research linking misinformation to vaccine hesitancy (Lin et al. 2020). At the time of the survey, all adults, regardless of comorbidity or immigration status, were eligible to receive the vaccine. However, only 69% of participants correctly believed they were eligible, while 22% were unsure and 8% incorrectly believed they were ineligible. Misconceptions about eligibility were uncorrelated with age, gender, and racial or ethnic background but exhibited a strong relationship with income. Individuals in households earning below $40,000 per year were 12% points less likely to correctly report that they were eligible for vaccination compared to those with higher incomes.

Participants expressed a range of concerns about the COVID‐19 vaccine, measured using a five‐point Likert scale. To mitigate question‐order bias, the order of concerns was randomized. The most frequently reported concern was vaccine safety, with 77% of respondents expressing concern (“agree” or “strongly agree”) about potential side effects. Additional concerns included fear of being forced to get vaccinated (64%), doubts about vaccine effectiveness (58%), and the belief that the vaccine itself could cause COVID‐19 infection (48%). Participants also reported concerns related to vaccine access, such as not knowing where to get vaccinated (29%) and worrying about paying for the vaccine (25%), despite widespread public messaging that vaccines were available free of charge.

The sample is balanced across control and incentive arms in terms of age, gender, race and ethnicity, household size, educational attainment, and concerns about the COVID‐19 vaccine. Table 1 presents baseline mean values in columns (2) and (3) for participants in the control and incentive arms, respectively, while column (4) reports p‐values comparing the means between the two groups. Additionally, an F‐test from a linear regression of treatment arm assignment on all baseline variables yields a p‐value of 0.33, indicating that overall differences between treatment arms are not statistically indistinguishable from zero. We conclude that random assignment was successfully implemented.

Finally, Table A1 compares our sample's baseline means with those of a representative sample of vaccine‐hesitant adults from the California Health Interview Survey (CHIS).^12^ Several factors contribute to the demographic differences between CHIS respondents and our analytical sample. First, although our survey was accessible via both phone and online modalities, nearly all participants opted for the online format. Prior research indicates that online respondents tend to be younger, more likely to be female, and more highly educated. Second, we deliberately oversampled non‐White participants to ensure sufficient statistical power for analyzing behavioral differences across racial and ethnic subgroups. Self‐reported health measures were comparable across both samples, with just over one in five respondents in each group reporting excellent health. Additionally, while CHIS samples respondents from across the entire state, our study was restricted to Los Angeles County. Despite this geographic difference, CHIS remains the most relevant survey for benchmarking our experimental sample.

## 4 Econometric Model

In this section, we develop an econometric model to examine individuals' willingness to get vaccinated under varying levels of monetary incentives. Following Pinto (2021), we observe three sets of variables: (i) a binary treatment assignment Z∈{0,1}; (ii) the incentive D(Z)∈{500,100,50,0}; and (iii) the vaccination decision Y(Z,D)∈{0,1}. In this model, an individual's choice to vaccinate depends on both the pecuniary benefit and any behavioral effects arising from exposure to the incentive.^13^ The choice equation is:

(5) $$Y=f_{Y}\left(Z,D,\epsilon \right)$$

where ϵ is an unobserved error term that is independent of Z.

The causal effect of a monetary incentive can be decomposed into the price effect and psychological effect, as reviewed in Section 2.2. The price effect unambiguously increases the attractiveness of vaccination, while the psychological effect has an opposing influence that may crowd out the incentivized behavior if it dominates the price effect. For illustration, consider an individual offered $500 to get vaccinated. The total effect on vaccine uptake is expressed as:

(6) $$\underset{\text{total}\;\text{effect}}{\underbrace{Y\left(1,500\right)-Y\left(0,0\right)}}=\underset{\text{price}\;\text{effect}}{\underbrace{Y\left(1,500\right)-Y\left(1,0\right)}}+\underset{\text{psychological}\;\text{effect}}{\underbrace{Y\left(1,0\right)-Y\left(0,0\right)}},$$

where Y(z,d) denotes the individual's vaccination decision under treatment assignment z and incentive d.

Previous studies have documented the existence of an adverse psychological effect by reporting negative total effects on an incentivized behavior, suggesting that the psychological effect can sometimes outweigh the price effect (Mellström and Johannesson 2008; Gneezy and Rustichini 2000; Frey and Jegen 2001). However, this test is overly conservative. As Equation (6) demonstrates, a non‐negative total effect does not imply that the psychological effect is zero. Rather, it indicates that, on average, the price effect weakly exceeds the psychological effect. In contrast, our experiment is designed to disentangle the price effect from the psychological effect by priming individuals to consider monetary incentives while simultaneously eliciting their willingness to get vaccinated when offered no payment.

To quantify the share of individuals deterred from vaccination (the “deterred share”), we classify participants by their latent response types, following approaches by Kline and Tartari (2016) and Heckman and Pinto (2018). A response type characterizes an individual's vaccination decision for every possible combination of treatment assignment z and incentive d. For instance, an always‐taker is defined as someone who vaccinates under all conditions: Y(z,d)=1∀(z,d). Although response types are unobservable, the statistical moments from our experiment can be expressed as mixtures of these latent response types, enabling us to derive policy‐relevant parameters as weighted averages over the response types. For clarity, consider the following response matrix in Equation (7), which organizes the response types $s_{i}$ alongside the corresponding choice behaviors $t_{j}$:

(7)

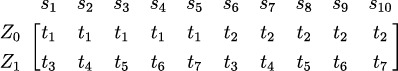

In the control arm $\left(Z_{0}\right)$, an individual with choice behavior $t_{1}$ chooses to vaccinate, while one with choice behavior $t_{2}$ does not. In the incentive arm $\left(Z_{1}\right)$, an individual with choice behavior $t_{3}$ vaccinates for any payment (including zero), $t_{4}$ vaccinates for no less than $50, $t_{5}$ vaccinates for no less than $100, $t_{6}$ vaccinates for no less than $500, and $t_{7}$ does not vaccinate for any payment.^14^

**FIGURE A5:**
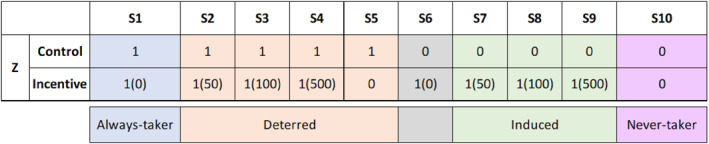
Treatment response types and their corresponding vaccination choices. Each column corresponds to one of 10 latent treatment response types, indicating whether an individual chooses to vaccinate (1) or not (0) under different control and incentive scenarios. For example, 1 (0) represents vaccination at zero payment, while 1 (50) indicates vaccination only when offered $50 or more. The color shading highlights four key response type categories: always‐takers (blue), deterred individuals (orange), induced individuals (green), and never‐takers (purple). Gray shading represents the monotonicity condition, which excludes the presence of {s6}.

In this model, response type $s_{1}\equiv \left(t_{1},t_{3}\right)$ denotes the always‐takers. Response types $\left\{s_{2},s_{3},s_{4},s_{5}\right\}$ denote deterred individuals: those who would vaccinate in the absence of any payment $\left(t_{1}\right)$ but reject the vaccine if primed with an incentive $\left(t_{4},t_{5},t_{6},t_{7}\right)$. Response types $\left\{s_{7},s_{8},s_{9}\right\}$ denote induced individuals: those who decline vaccination without payment $\left(t_{2}\right)$ but accept the vaccine for some positive payment $\left(t_{4},t_{5},t_{6}\right)$. Finally, response type $s_{10}\equiv \left(t_{2},t_{7}\right)$ denotes the never‐takers. Figure A5 illustrates these response types.

Here, we formalize a monotonicity condition to nonparametrically identify the always‐taker share and the deterred share. This monotonicity condition eliminates response type $s_{6}\equiv \left(t_{2},t_{3}\right)$, in the same spirit that Imbens and Angrist (1994) rule out defiers to identify the local average treatment effect. Our monotonicity assumption states that no individual is induced to get vaccinated by a zero‐payment incentive (after being primed to consider incentives) relative to the control arm, which is not primed with any incentive. This assumption follows from 2 decades of theoretical and empirical behavioral research, which demonstrates that, after accounting for the price effect, extrinsic incentives unambiguously weaken intrinsic motivation for free‐choice behavior (Frey and Oberholzer‐Gee 1997; Bowles and Polania‐Reyes 2012).

How does our monotonicity condition identify the always‐taker share and the deterred share? Our argument proceeds as follows. First, the set of always‐takers plus deterred types, $\left\{s_{1},s_{2},s_{3},s_{4},s_{5}\right\}$, is identified by participants in the control arm who choose to vaccinate. Because treatment assignment Z is randomly assigned, this share is equivalent between the control and incentive arms. Next, under the monotonicity condition, we rule out vaccinations induced by a zero‐payment incentive, $\left\{s_{6}\right\}$. Consequently, we identify always‐takers, $\left\{s_{1}\right\}$, as individuals in the incentive arm who vaccinate when offered no payment. Finally, subtracting always‐takers from the combined always‐taker plus deterred set yields the share of deterred types, $\left\{s_{2},s_{3},s_{4},s_{5}\right\}$.

## 5 Main Results

### 5.1 Impact on Willingness to Vaccinate

**FIGURE 1:**
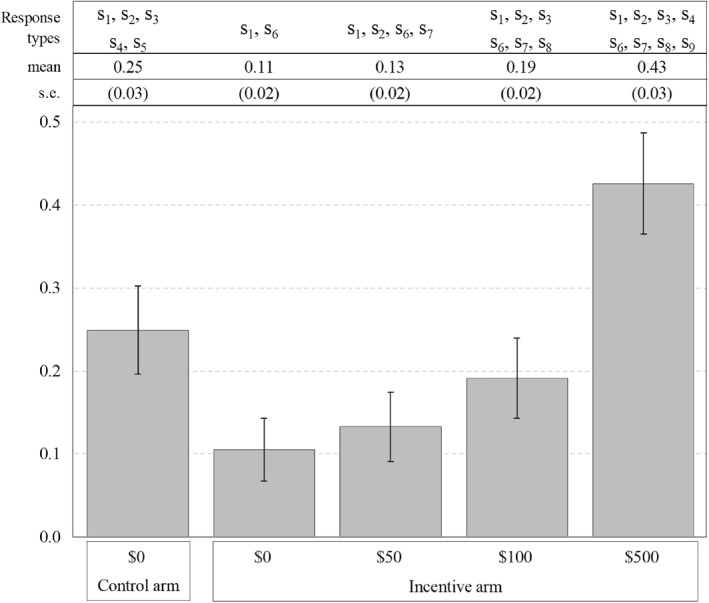
Willingness to vaccinate stratified by treatment arm and incentive amount. This figure presents estimates of individuals' willingness to get vaccinated against COVID‐19, stratified by treatment assignment and incentive amount. The figure distinguishes between the control arm, where no financial incentive was provided, and the incentive arm, where participants were offered monetary incentives ranging from $0 to $500. The y‐axis represents the proportion of individuals who expressed a willingness to receive the COVID‐19 vaccine. The figure presents 95‐percent confidence intervals from robust standard errors. Above the figure, the table summarizes the response type mixtures associated with each estimate. The first row labels the response types included in each category, while the second row presents the mean estimates. The third row reports robust standard errors in parentheses.

This section estimates the causal effect of being primed to consider an incentive on individuals' willingness to get vaccinated. Figure 1 presents our main findings. Differences between the control and incentive arms are estimated using a linear regression of stated willingness to vaccinate on the incentive‐arm indicator, with robust standard errors. In the control arm, 25% of participants reported a willingness to vaccinate without any payment. By contrast, in the incentive arm, willingness varied by the offered payment: 43% at $500, 19% at $100, 13% at $50%, and 11% at zero payment. Section 4 details how these statistics map onto the underlying response types: always‐takers, deterred individuals, induced individuals, and never‐takers. For instance, participants in the control arm who were willing to vaccinate (25%) must belong to one of the five response types $\left\{s_{1},s_{2},s_{3},s_{4},s_{5}\right\}$.

We decompose the response‐type mixture representing the 25% of participants willing to get vaccinated in the control arm into two distinct groups. Recall that these participants must be either always‐takers $\left(s_{1}\right)$ or deterred individuals $\left(s_{2},s_{3},s_{4},s_{5}\right)$. Our identification exercise indicates that 10.5% (s.e.=1.9) of participants are always‐takers; they would get vaccinated in the incentive arm even when offered zero payment. Consequently, we deduce that 14.4% (s.e.=3.3) of participants are in the deterred group; they would vaccinate without any payment but reject vaccination if offered a nonzero incentive.

While the deterred share and always‐taker share are separately point identified, the never‐taker share, $\left\{s_{10}\right\}$, and induced share, $\left\{s_{7},s_{8},s_{9}\right\}$, are not. In particular, individuals who reject vaccination even when offered $500 might not be never‐takers if, for them, the adverse psychological effect outweighs the price effect. However, since the probabilities of all response types must sum to one, these shares are partially identified. Following the linear programming framework developed by Honoré and Lleras‐Muney (2006), we estimate bounds for these parameters by treating the identified response‐type mixtures as constraints within an optimization problem. By construction, these bounds include every value consistent with our experimental data and the monotonicity condition, yielding estimated bounds for the never‐taker share of [43%, 57%] and the induced share of [18%, 32%].^15^

### 5.2 Who Gets Deterred by Incentives?

Heterogeneous responses to a vaccine incentive may arise from differences in past experiences with the health care system or varying levels of trust in institutions. For instance, marginalized groups in the U.S. have a history of negative encounters with health care providers, such as the Tuskegee Syphilis experiment, which has been linked to persistently lower health care utilization among Black men (Alsan and Wanamaker 2018). To investigate whether the psychological effect induced by an incentive differs across demographic subgroups, we use baseline survey data to characterize participants who were deterred from vaccination. Our approach employs a nonparametric κ‐weighting scheme formalized by Abadie (2003) for estimating statistics for the complier population. We derive estimates for the always‐taker and deterred subpopulations in our experiment.

Table 2 reports mean baseline characteristics for deterred individuals and always‐takers, who comprise 14% and 11% of our sample, respectively. Statistics for these groups are separately point identified. Column (3) presents individual p‐values testing for mean differences between them. We detect substantial heterogeneity in psychological effects by race and gender. Notably, non‐White men account for one‐third of the deterred population but only 4% of the always‐taker population (p=0.014). No other demographic, including household size or age, exhibits a disparity of this magnitude. Thus, race and gender appear to be salient factors influencing how individuals perceive and respond to a vaccine incentive.

**TABLE 2:** Baseline demographics and vaccine concerns by treatment response type.

	(1)	(2)	(3)
	Deterred	Always‐taker	p‐value
A. Participant demographics			
Household size	4.16	3.89	0.66
	(0.46)	(0.26)	
Age	28.8	33.4	0.34
	(2.8)	(2.5)	
White male	0.11	0.19	0.61
	(0.09)	(0.07)	
White female	0.16	0.30	0.45
	(0.11)	(0.09)	
Non‐white male	0.33	0.04	0.01
	(0.10)	(0.04)	
Non‐white female	0.38	0.48	0.60
	(0.13)	(0.10)	
B. Vaccine concerns (1–5 scale)			
Side effects	4.4	3.5	0.04
	(0.3)	(0.2)	
Not effective	3.7	3.5	0.60
	(0.3)	(0.2)	
Causes infection	3.2	3.6	0.36
	(0.3)	(0.2)	
Affordability	2.7	3.1	0.46
	(0.4)	(0.2)	
Compulsion by law	3.6	3.3	0.49
	(0.3)	(0.2)	
Accessibility	3.1	3.2	0.79
	(0.3)	(0.2)	
Compulsion by work/school	3.6	3.5	0.78
	(0.3)	(0.2)	
Observations	74	54	

*Note:* This table presents mean values for baseline variables, stratified by participants' treatment response type. Panel A reports demographic characteristics, while Panel B presents pre‐treatment concerns about the COVID‐19 vaccine, measured on a 1‐5 point Likert scale, where higher values indicate greater concern. Column (1) displays means and standard errors for deterred individuals, represented by treatment response types {$s_{2},s_{3},s_{4},s_{5}$}, while Column (2) presents statistics for always‐takers, {$s_{1}$}. Column (3) two‐sided, single‐hypothesis p‐values from tests of mean equality between deterred individuals and always‐takers. Treatment response types were identified using Abadie's (2003) κ method, which recovers the proportions of response types in the population.

Additionally, baseline concerns about the vaccine's side effects differ substantially between the deterred and always‐taker populations. Deterred individuals express significantly higher levels of distrust in the vaccine's safety compared to always‐takers, with an average of 4.4 versus 3.5 on the 1‐5 Likert scale (p=0.041). However, the two groups do not differ significantly in their baseline concerns about the vaccine's efficacy or affordability.

### 5.3 Anchoring Bias

As noted in Section 3.1, any estimate of an adverse psychological effect must be interpreted relative to the initial $500 value presented in the incentive arm. This reflects anchoring bias: large, salient numbers disproportionately shape subsequent valuations (Lieder et al. 2018). Importantly, anchoring does not threaten the internal validity of our causal estimates, as randomization ensures that treatment effects are unconfounded. Rather, any effect should be understood with respect to the anchor value. In our context, anchoring bias may be more influential among lower‐income respondents, for whom $500 represents a larger share of their income and is thus more likely to shape vaccination intentions. If the deterrent effect is concentrated in this subgroup, then anchoring bias may have introduced heterogeneity in our experiment.

To assess this possibility, we estimate a linear regression of stated willingness to vaccinate on the incentive‐arm indicator, a dummy variable for household income below $40,000 (“low income”), and their interaction, with robust standard errors. Roughly half of the sample is classified as low income. Regression results are presented in Table A2. The coefficient on the incentive‐arm indicator is −0.13, consistent with our main finding that exposure to the incentive reduces willingness to vaccinate. The main effect of low income is positive but statistically insignificant (0.04, p=0.41). Crucially, the interaction between treatment assignment and low income is small (−0.03) and statistically insignificant (p=0.61). Results from this sensitivity analysis imply that the incentive's deterrent effect does not vary across income strata. Instead, the adverse psychological response was broadly shared across income groups, reinforcing our interpretation that a monetary incentive undermines intrinsic motivation, rather than reflecting an anchoring bias tied to the incentive's absolute size.

## 6 Mediation Analysis

Our experiment examines two mechanisms through which a monetary incentive might discourage vaccination. The first is the signaling effect: does the incentive imply that the vaccine carries greater risks? Prior research suggests that clinical studies offering large payments are perceived as riskier than those offering no payment (Cryder et al. 2010). The second mechanism involves the crowding out of prosocial motives. Many vaccination campaigns emphasize social responsibility, but individuals who view vaccination as an altruistic act may no longer perceive it as such when primed with an incentive. This shift occurs because pecuniary and prosocial motives can be difficult to reconcile (Frey and Jegen 2001; Akerlof and Kranton 2010).

To identify these mechanisms, we developed a mediation framework that examines both channels simultaneously. Since our experiment introduces exogenous variation in exposure to an incentive, we estimate its causal impact on participants' beliefs about the COVID‐19 vaccine. Participants reported whether they believed the vaccine was safe, unsafe, or if they were unsure. They provided analogous responses regarding whether getting vaccinated is a social responsibility (see Section 3.1). We define four mediators based on their responses to these questions: $M_{1}$ measures beliefs that the vaccine is unsafe, $M_{2}$ measures uncertainty about the vaccine's safety, $M_{3}$ measures beliefs that vaccination is not a responsibility, and $M_{4}$ measures uncertainty about whether vaccination is a responsibility.

For mediator $M_{j}$ to serve as a link between the incentive (Z) and an individual's willingness to vaccinate (Y), the incentive must affect the mediator via $\gamma_{j}$ and, in turn, the mediator must affect one's willingness to vaccinate via $\theta_{j}$. We formalize this in Equation (8), which decomposes the overall effect of the psychological effect into a direct effect (Δ) and the indirect effects $\left(\sum \gamma_{j}\theta_{j}\right)$:

(8)

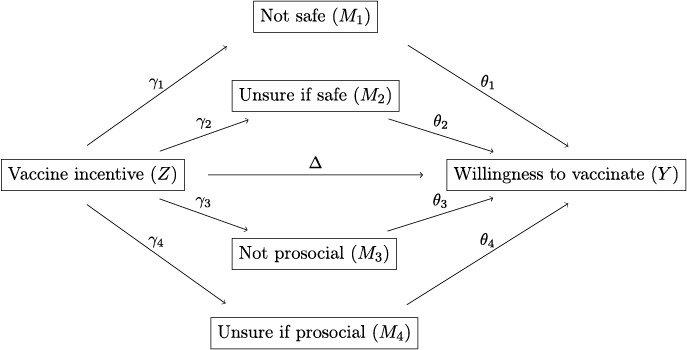




Table A3 reports mean values of the mediator variables, as well as their cross‐tabulation. In the full sample, 46% of respondents considered the vaccine unsafe, 43% were unsure about its safety, 54% did not report prosocial motivations, and 24% were unsure about prosociality. Among those who viewed the vaccine as unsafe $\left(M_{1}=1\right)$, 72% also reported not being prosocial. Respondents who were unsure about prosociality $\left(M_{4}=1\right)$ were more likely to report uncertainty about vaccine safety (66%). Overall, we observe a strong alignment between safety concerns and weaker prosocial motivations to vaccinate.

**FIGURE 2:**
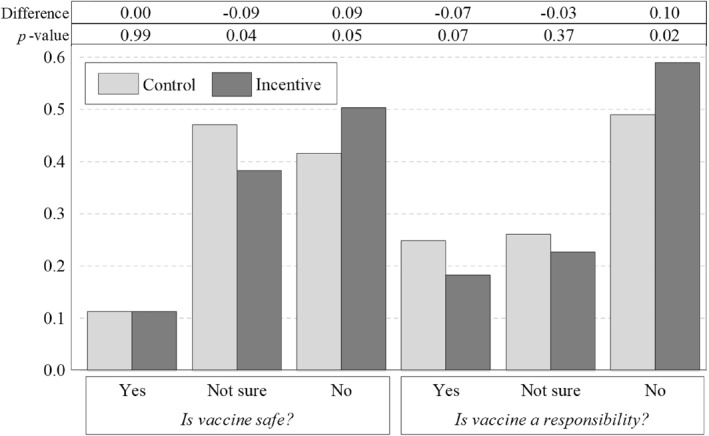
Differences in perceptions about the COVID‐19 vaccine by treatment arm. This figure presents estimates of individuals' perceptions regarding the COVID‐19 vaccine's safety (“Do you believe the coronavirus vaccine is safe?”) and prosocial attitudes toward vaccination (“Do you believe people have a responsibility to get vaccinated for the coronavirus?”), disaggregated by treatment arm assignment. The y‐axis indicates the proportion of respondents responding “yes,” “not sure,” or “no.” The control arm is represented in light gray, while the incentive arm is in dark gray. Percentage‐point differences between the incentive and control arms are displayed, along with two‐sided, single‐hypothesis p‐values assessing the equality of estimates between the two arms.

Figure 2 presents the reduced‐form results of our mechanisms analysis. Participants randomized to the incentive arm were 9% points more likely to believe that the vaccine is unsafe compared to those in the control arm (50% vs. 41%). A gap of the same magnitude, but opposite in sign, appeared in the proportion of participants who reported being “unsure” about the vaccine's safety (38% vs. 47%), suggesting that the incentive primarily influenced those with uncertainty in their beliefs. In contrast, participants in the incentive arm were neither more nor less likely to believe the vaccine was safe than those in the control arm.

Similarly, participants in the incentive arm were 10% points more likely to report that people do not have a responsibility to get vaccinated (59% vs. 49%). Two‐thirds of this effect stems from a decline in the share of participants who believe vaccination is a responsibility (18% vs. 25%), while the remaining third comes from a decrease in those expressing uncertainty about this belief (23% vs. 26%).

To estimate the parameters in Equation (8), we regress willingness to get vaccinated (Y) on the set of mediators $\left(M_{i}\right)$ and the treatment indicator (Z), while also regressing each mediator on the treatment indicator. The coefficients are estimated simultaneously to account for the pathways' joint determination, with standard errors adjusted accordingly. Specifically, parameters were estimated using generalized structural equation modeling, which accommodates multiple mediation pathways within a single framework. Parameters were estimated via maximum likelihood with robust standard errors, consistent with Equation (8).

Table 3 reports the estimated coefficients for Δ, γ, and θ. This mediation model decomposes the psychological effect into the direct effect (Δ) and indirect effects $\left(\sum \gamma_{j}\theta_{j}\right)$. The indirect effects represent the portion explained by shifts in participants' perceptions of vaccine safety and their prosocial attitudes toward vaccination. These mediators account for 27% of the adverse psychological effect. The empirical results also reinforce our motivating theoretical framework, which help us interpret the observed psychological mechanisms. We find that monetary incentives reduced respondents' stated intentions to vaccinate, consistent with a setting in which both risk signaling (ρ) and prosocial crowd‐out (δ) are positive for a meaningful share of individuals.

**TABLE 3:** Estimated coefficients of the mediation model.

	Dependent variable				
	Y	$M_{1}$	$M_{2}$	$M_{3}$	$M_{4}$
Incentive arm (Z)	−0.11	0.09	−0.09	0.10	−0.03
	(0.03)	(0.04)	(0.04)	(0.04)	(0.04)
Not safe $\left(M_{1}\right)$	−0.41				
	(0.07)				
Unsure if safe $\left(M_{2}\right)$	−0.31				
	(0.07)				
Not prosocial $\left(M_{3}\right)$	−0.29				
	(0.05)				
Unsure if prosocial $\left(M_{4}\right)$	−0.21				
	(0.06)				
$R^{2}$	0.31	0.01	0.01	0.01	0.00
Observations	513	513	513	513	513

*Note:* This table presents ordinary least squares estimates from the causal mediation model described by Equation (8). The dependent variable, Y, is an indicator equal to one if a participant considered getting vaccinated against COVID‐19. The treatment variable, Z, equals one for participants randomly assigned to the incentive arm of the experiment. The mediators, $\left\{M_{1},M_{2},M_{3},M_{4}\right\}$, capture participants' perceptions of vaccine safety and their prosocial attitudes toward vaccination, which were measured after participants were randomized within the experimental module. The coefficients were estimated simultaneously to account for the joint determination of the mediation pathways. Robust standard errors, which account for simultaneous estimation, are reported in parentheses.

## 7 Qualitative Evidence

We conducted a mixed‐methods analysis nested within our broader survey experiment (Creswell and Clark 2018). To do this, a subset of participants (N=27) was recruited for 30‐min, semi‐structured interviews.^16^ The interviews focused on three domains: concerns and distrust toward the COVID‐19 vaccine; desires for policy interventions including monetary incentives; and preferences for non‐pharmacological interventions. Transcripts were analyzed thematically through iterative codebook development, comparisons between codes and transcripts, and consensus decisions on emergent themes. Inter‐rater reliability testing yielded a κ statistic of 0.78, indicating substantial team agreement in coding transcripts.^17^

The qualitative findings from the interviews align with this paper's quantitative finding that the presence of a monetary incentive generated negative perceptions of COVID‐19 vaccines. In particular, incentives heightened suspicion toward vaccine effectiveness and safety among vaccine‐hesitant participants. Non‐monetary incentives, such as free food or event tickets, elicited similar reactions, though they were not directly tested in our survey experiment. Below, we present exemplar quotes from participants:

The money incentives to get the vaccines, my friend was extremely suspicious of that. Because she says, if it needs to be incentivized that way, it makes her even more unwilling to get the vaccine because it sounds more like a pushing agenda or why do they need to kind of, not trick people, but incentivize them that way? Like, this seemed less scientific, but more of a hidden agenda behind it.

25 year old, Asian

The way they're going about trying to get people to get is like just sketchy. I don't think in history or during my time being here I've ever seen them try to. Like, for example, you get a free Krispy Creme donut if you have the vaccine. I've seen the other day, they were like there were something involving like a lottery ticket.

19 year old, Black

At the store down the street, they they'll give you $50 if you get vaccinated and to me I don't know that's like to me, that's on the lines of like selling yourself for people to test [you]. I don't know. I think…I, I just, I don't like it.

44 year old, White

I just feel like it became a little weird when with the whole lottery, like they're giving away money and things like that just to kind of get people to want to go ahead and get the vaccine…I actually would be more likely [without the lottery] because, like I said, I will go to my peers and ask them questions to find out more so information on that versus getting something for it. They may not necessarily be thinking of their health, or, you know, at the moment, they're moreso just thinking about the money.

31 year old, American Indian

From our analysis of the qualitative interviews, four key themes emerged. First, public health overcommunication can be a hindrance and may contribute to distrust. Several respondents described feeling skeptical when confronted with repeated messaging, which in turn heightened suspicion of public health authorities. Second, unvaccinated individuals' attitudes toward vaccines fall along a wide continuum. Rather than fitting neatly into “pro‐” or “anti‐” vaccine categories, respondents expressed a spectrum of views ranging from mild uncertainty to strong opposition. Relatedly, many vaccine‐hesitant individuals reported a preference for non‐pharmacological interventions, such as masking and physical distancing. Third, bodily autonomy emerged as a central principle. Participants frequently framed their vaccination choices in terms of control over their own health and bodies, often invoking the theme of “my body, my choice.” Finally, numerous verbatim responses illustrate psychological mechanisms consistent with reactance, a defensive response that arises when individuals perceive their autonomy as constrained (Brehm and Brehm 2013). The first and second verbatim, in particular, capture the concern that monetary incentives may signal manipulation rather than support informed choice. Although these four themes extend beyond the scope of our quantitative experimental aims, they provide valuable context for interpreting our main findings.

## 8 Discussion

In this paper, we show that a monetary incentive can undermine individuals' willingness to get vaccinated against COVID‐19. In our novel survey experiment, 14% of participants who were initially willing to consider vaccination without any payment were deterred when primed with an incentive. This crowd‐out effect is economically meaningful, given that only one‐quarter of respondents expressed willingness at baseline. Notably, those who were deterred tend to be men from racial and ethnic minority backgrounds and exhibited, at baseline, heightened concerns about vaccine side effects, indicating that incentives do not uniformly increase or decrease uptake. The effect is indeed heterogeneous. Finally, our mediation model traces this result to worsened perceptions of vaccine safety and weakened prosocial attitudes toward vaccination, which together explain roughly 25% of the negative psychological effect.

One limitation of our study is that we do not measure realized vaccination behavior, focusing instead on participants' stated willingness to get vaccinated. Although intention is a necessary precursor to action, this measure does not capture the intention‐to‐behavior gap. This limitation is particularly relevant for the vaccine‐hesitant population, as individuals who express skepticism may not follow through on their stated intentions due to external factors such as changing public health messaging, social influence, or evolving perceptions of vaccine safety over time. While our findings provide insights into the motivational factors influencing vaccine hesitancy, future research should incorporate our methodological approach while tracking actual vaccine uptake to extend these findings.

In addition, our mediation analysis examined only two psychological mechanisms (i.e., perceptions of vaccine safety and social responsibility), which together account for one‐quarter of incentives' adverse psychological effect. While this share is large, the majority remains unexplained. Another plausible pathway is psychological reactance, the defensive response that arises when individuals perceive their freedom of choice to be threatened. Monetary incentives may trigger such reactance by implying that individuals would not otherwise choose vaccination freely. Although we did not directly test for this mechanism using our experiment, qualitative evidence provide suggestive evidence for it.

Another limitation is that we cannot disentangle an incentive's priming effect from the anchoring effect. Since participants in the incentive arm were first asked about a $500 payment, their subsequent responses may have been influenced by this value as an anchor, shaping their willingness to vaccinate at a lower payment. Our research design allowed us to isolate the adverse psychological effect of introducing a monetary incentive, but the anchoring effect may have amplified (or muted) this effect. This limitation cannot be remedied ex post. We note, however, that $500 fell within the range of real‐world proposals under discussion by U.S. policymakers and employers at the time of the pandemic, making it a policy‐relevant reference point.

From a methodological perspective, our results challenge a core assumption in many experimental designs: that monetary incentives encourage treatment uptake. Often, in order to estimate a causal treatment effect, researchers randomly allocate incentives for individuals to accept a treatment, assuming that incentives weakly push individuals toward treatment take‐up. For instance, Thornton (2008) randomly allocated payments for rural Malawians to learn their HIV status, using these incentives as an instrumental variable. In our context, however, the “no defiers” assumption breaks down; one in seven were dissuaded by the prospect of payment. This subgroup would receive negative weight in the two‐stage least squares estimator. For interventions where incentives may discourage uptake, researchers should consider weaker forms of monotonicity or directly test for defiers using our procedure or others (Kitagawa 2015; Mourifié and Wan 2017; Christy and Kowalski 2024).

Finally, our findings have direct policy implications for the design of public health interventions to increase vaccination uptake. Policymakers should exercise caution when using monetary incentives, as these may backfire among certain subpopulations, particularly racial and ethnic minorities and those who already exhibit higher levels of vaccine hesitancy. Rather than relying solely on monetary payments, campaigns should emphasize vaccine safety and prosocial motives to avoid framing vaccination as a transactional decision, which risks eroding trust in public health. In particular, direct cash payments may be more likely to signal risk or crowd out intrinsic and prosocial motivations. By contrast, symbolic incentives or unconditional supports, such as covering transportation or time costs, may achieve greater acceptability without the same psychological drawbacks. Future research should directly compare the relative effectiveness of monetary versus symbolic, and conditional versus unconditional, incentives.

In the short run, a monetary incentive's effectiveness depends on its net impact: how many people does the incentive persuade and how many does it incentive dissuade? This can explain why an incentive succeeds in some settings (Campos‐Mercade et al. 2021) but fails in others (Chang et al. 2021). In the long run, however, a monetary incentive can erode trust in public health institutions. Notably, we find that exposure to the idea of an incentive reduces the share of participants who believe that vaccination is a social responsibility. Pandemic mitigation strategies, such as vaccination, mask mandates, and social distancing, are only effective if people adhere to them and assume responsibility for controlling the spread of the disease. Monetary incentives may backfire in this regard.

## Consent

We confirm that this research complies with ethical guidelines. The study protocol was approved by the Institutional Review Board at UCLA (IRB# 21‐000219), and all participants provided informed consent.

## Conflicts of Interest

The authors declare no conflicts of interest.

## Data Availability

The data that support the findings of this study are available on request from the corresponding author. The data are not publicly available due to privacy or ethical restrictions.

## Endnotes

^1^
Conditional cash transfer programs, such as Brazil's *Bolsa Família* and Mexico's *Prospera*, tie financial payments to school attendance and routine health check‐ups. These requirements aim to promote human capital accumulation and adherence to preventive health measures, and have been shown to improve educational and health outcomes for children (IMF 2019).

^2^
While the U.S. federal government never experimented with monetary incentives to boost COVID‐19 vaccine uptake, several states, such as Ohio and West Virginia, offered lotteries and $100 bonds to incentivize vaccination, respectively. Some private companies also paid employees to get vaccinated (Krouse 2021).

^3^
For instance, a field experiment found that offering monetary compensation for blood donation reduced the likelihood of women donating blood, suggesting that extrinsic rewards can undermine intrinsic motivation (Mellström and Johannesson 2008). Similarly, high school students raised less money for charity when paid a commission to do so (Gneezy and Rustichini 2000). For a review of the literature on the adverse impacts of monetary incentives, see Gneezy et al. (2011).

^4^
Similarly, evaluations of incentives for other vaccines have produced mixed results (Wigham et al. 2014; Tressler and Bhandari 2019; Mantzari et al. 2015; Banerjee et al. 2010; Sato and Fintan 2020). One systematic review found that incentive‐based interventions were among the least effective strategies for increasing vaccine uptake (Jarrett et al. 2015).

^5^
This initiative also streamlined regulatory processes by enabling emergency‐use authorizations based on accelerated clinical trial designs, shorter follow‐up periods, and reduced data requirements. By doing so, it effectively shared both financial risk and scientific innovation across the public and private sectors.

^6^
We used convenience sampling through both recruitment channels. First, our research team partnered with nine community‐based nonprofit organizations to disseminate the survey to their constituents, using social media flyers and organizational networks to enhance engagement. This approach has been shown to reach individuals who may be less accessible through traditional recruitment methods (Nguyen et al. 2010). Second, we partnered with Qualtrics, which identified potential respondents residing in Los Angeles County.

^7^
Randomization was conducted within Qualtrics software using its built‐in randomization function. We verify baseline covariate balance between the control and incentive arms in Section 3.2.

^8^
At the time of our survey launch, three COVID‐19 vaccines had been approved for emergency use by the U.S. Food & Drug Administration. To reduce the questions' cognitive load, we did not ask participants to differentiate between the COVID‐19 vaccine brands.

^9^
At the time of survey design, $500 was discussed in U.S. policy debates as a plausible incentive level for COVID‐19 vaccination, making it a salient and policy‐relevant benchmark for respondents. Second, we aimed to calibrate the anchor at a level high enough to be credible, yet not so extreme as to appear unrealistic. Importantly, our analysis does not assume that $500 is an optimal or preferred incentive.

^10^
To minimize response bias, the vaccine safety and prosocial responsibility questions were presented as part of a broader set of survey questions on health beliefs.

^11^
Our analytical sample excluded 10 respondents (1.9%) who completed the survey in fewer than 3 minutes, as extremely short completion times indicate inattentive or low‐quality responses.

^12^
CHIS is a comprehensive health survey conducted annually by the UCLA Center for Health Policy Research in collaboration with the California Department of Public Health and the Department of Health Care Services. It uses a stratified sampling methodology and both online and telephone modalities to capture health‐related data. For our analysis, we stratified the CHIS sample based on responses to the question: “If a vaccine becomes available for COVID‐19, would you get it?” In this stratification, 4183 individuals responded “no,” which, after applying survey weights, corresponds to an estimated 7 million vaccine‐hesitant adults across California.

^13^
Although Z is randomized, it does not act as a conventional instrumental variable because Z influences the outcome Y beyond its effect on the incentive D. That is, the psychological effect violates the exclusion restriction. Nonetheless, it meets the independence and relevance conditions typically required of instrumental variables.

^14^
For tractability, we assume that individuals are “money rational,” meaning that no participant in the incentive arm who rejects the vaccine for $500 would subsequently accept it for $100 or less. We validate this assumption by demonstrating that the overall relationship between monetary incentives and vaccine willingness is monotonic (Figure 1). This assumption simplifies our econometric model by reducing the number of possible response types from 64 to 10.

^15^
We implement Mehrotra's (1992) predictor‐corrector primal‐dual method to solve our constrained optimization problem. This approximation‐based approach makes solving large problems more feasible than the simplex algorithm and is also more computationally efficient.

^16^
Participants completed interviews via Zoom or telephone (23 in English and 4 in Spanish) with one of four qualitatively trained analysts on our research team. Interviews were recorded, translated into English if needed, and transcribed.

^17^
This analysis adapted Watkins' (2012) team analysis approach for qualitative data. Our coding process involved five coders, including the four interviewers. First, we revisited the aims of the study to ensure alignment with our analysis. To familiarize ourselves with the data, we randomly selected three transcripts for the team to read together. Each coder then reread these transcripts independently and generated preliminary codes that captured the main ideas. Next, as a group, we reviewed and compared codes, refining them through consensus to develop a codebook with operational definitions. After completing the coding process, the group reconvened to discuss emergent themes.

## Appendix A

**TABLE A1:** Comparison of baseline statistics with vaccine‐hesitant adults in California.

Would get COVID‐19 vaccine?	California health interview survey		Our sample
	Yes	No	No/not sure
Age			
18–34	0.30	0.32	0.55
35–49	0.24	0.27	0.33
50–64	0.23	0.25	0.08
65 and over	0.23	0.16	0.04
Man	0.51	0.44	0.34
Woman	0.49	0.56	0.65
Race and ethnicity			
Latino	0.36	0.50	0.38
Asian	0.15	0.08	0.09
Black	0.04	0.10	0.35
White	0.41	0.29	0.34
College graduate	0.46	0.26	0.32
Ever tested positive	0.14	0.13	0.12
Self‐reported health			
Excellent	0.20	0.21	0.23
Very good	0.35	0.31	0.35
Good	0.30	0.32	0.28
Fair	0.13	0.13	0.11
Poor	0.02	0.03	0.03
Survey N	17,766	4183	513
Population N	22,727,828	6,957,054	

*Note:* This table compares baseline variable means between our analytical sample and respondents from the 2020 California Health Interview Survey (CHIS; Adult Survey). While CHIS samples respondents from across California, our study focuses on residents of Los Angeles County. Despite this geographic difference, CHIS remains the most relevant survey for contextualizing our sample. The first column reports mean values for CHIS respondents who answered “yes” to the question, “If a vaccine becomes available for COVID‐19, would you get it?” The second column reports mean values for CHIS respondents who answered “no” to this question. The third column presents mean values for participants in our survey experiment. CHIS estimates were weighted using strata and cluster weights to ensure representativeness of the California population.

**TABLE A2:** Estimated coefficients for the anchoring bias regression.

	Main model	Interacted model
Incentive arm (Z)	−0.14	−0.13
	(0.03)	(0.05)
{Income < $40,000}		0.04
		(0.05)
Z× {income < $40,000}		−0.03
		(0.07)
Intercept	0.25	0.23
	(0.03)	(0.04)
Observations	513	513

*Note:* This table reports ordinary least squares estimates from the regression analysis specified in Section (5.3). The dependent variable, Y, is an indicator equal to one if a participant considered getting vaccinated against COVID‐19. The treatment variable, Z, equals one for participants randomly assigned to the incentive arm. The indicator variable {Income < $40,000} equals one if the respondent's stated annual household income was less than $40,000. Robust standard errors are shown in parentheses.

**TABLE A3:** Contingency table for mediator variables.

	Subsample				
	Full sample	$M_{1}=1$	$M_{2}=1$	$M_{3}=1$	$M_{4}=1$
Not safe $\left(M_{1}\right)$	0.46			0.61	0.28
Unsure if safe $\left(M_{2}\right)$	0.43			0.34	0.66
Not prosocial $\left(M_{3}\right)$	0.54	0.72	0.43		
Unsure if prosocial $\left(M_{4}\right)$	0.24	0.15	0.38		
Observations	513	236	219	277	125

*Note:* This table presents a contingency table cross‐tabulating the variables from the causal mediation model described in Equation (8). Each entry reports the mean of the corresponding variable, either for the full sample or for subsamples defined by the values of other mediators. The mediators, $\left\{M_{1},M_{2},M_{3},M_{4}\right\}$, capture participants' perceptions of vaccine safety and their prosocial attitudes toward vaccination, which were measured after participants were randomized within the experimental module.
